# Effectiveness of shared goal setting and decision making to achieve treatment targets in type 2 diabetes patients: A cluster‐randomized trial (OPTIMAL)

**DOI:** 10.1111/hex.12563

**Published:** 2017-05-24

**Authors:** Henk Den Ouden, Rimke C. Vos, Guy E. H. M. Rutten

**Affiliations:** ^1^ Julius Center for Health Sciences and Primary Care University Medical Center Utrecht Utrecht The Netherlands

**Keywords:** shared decision making, type 2 diabetes, treatment targets

## Abstract

**Objective:**

About 20% of patients with type 2 diabetes achieve all their treatment targets. Shared decision making (SDM) using a support aid based on the 5‐years results of the ADDITION study on multifactorial treatment, could increase this proportion.

**Research design and methods:**

Cluster‐randomized trial in 35 former ADDITION primary care practices. Practices were randomized to SDM or care as usual (1:1). Both ADDITION and non‐ADDITION type 2 diabetes patients, 60‐80 years, known with diabetes for 8‐12 years, were included. In the intervention group, patients were presented evidence about the relationship between treatment intensity and cardiovascular events. They chose intensive or less intensive treatment and prioritized their targets. After 1 year priorities could be rearranged. Follow‐up: 24 months. Intention‐to‐treat analysis. Main outcome measure: proportion of patients that achieved all three treatment targets.

**Results:**

At baseline 26.4% in the SDM group (n=72) had already achieved all three treatment goals (CG: 23.5%, n=81). In the SDM group 44 patients chose intensive treatment, 25 continued their former less intensive treatment and three people switched from the more to the less intensive protocol. After 24 months 31.8% of the patients in the SDM group achieved all three treatment targets (CG: 25.3%), RR 1.26 (95% CI 0.81‐1.95). Mean systolic blood pressure decreased in the SDM group (−5.4 mm Hg, *P*<.01), mean HbA1c and total cholesterol did not change.

**Conclusions:**

Despite an already high baseline level of diabetes care, we found strong indications that SDM on both intensity of treatment and prioritizing treatment goals further improved outcomes.

## 1 INTRODUCTION

The control of type 2 diabetes mellitus (T2DM) involves a complex series of medical decisions with respect to treatment goals, self‐care behaviours and medical treatments.^1^, ^2^ It requires frequent follow‐up visits with reconsidering treatment priorities and patients' preferences.^2^, ^3^ The quality of these decisions could influence the appropriate treatment of T2DM.^2^, ^3^, ^4^, ^5^ Adequate treatment of multiple risk factors can prevent or postpone diabetes related complications.^1^, ^6^, ^7^, ^8^

In practice about 10%‐20% of patients with T2DM achieve all treatment targets for glycemic control, lipids and blood pressure,^9^, ^10^ whereas reported percentages for separate targets are much higher (30%‐70%).^11^, ^12^, ^13^, ^14^ Clinicians are sometimes hesitant to intensify treatment,^11^, ^15^ and patients are not always adherent to medical treatment,^16^, ^17^ and doctors do not acknowledge this.^11^, ^16^, ^17^ A collaborative approach by using shared decision making (SDM) and goal setting could be helpful for both patient and clinician and might increase treatment adherence and the proportion of patients who successfully reach all their treatment targets.^1^, ^18^, ^19^, ^20^, ^21^, ^22^

Shared decision making is an approach that respects the clinical evidence and patient's preferences for treatment goals. SDM is defined as “an approach where clinicians and patients make decisions together, using the best available evidence”.^18^ It promotes patient's involvement in weighting benefits and harms of evidence‐based treatment options.^18^, ^19^ Shared goal setting is defined as the agreement between health‐care professionals and patients on health‐related goals.^20^, ^21^, ^22^

The quality of diabetes care with integration of SDM and goal setting could be enhanced by a personalized decision aid, that takes into account both the patient's clinical characteristics as well as treatment preferences.^23^, ^24^, ^25^, ^26^ Decision aids are proven effective in involving the patient in the shared decision‐making process.^25^ During the last decade such aids were developed to support the achievement of patient‐centred treatment goals and options for lifestyle modifications and medication use.^25^, ^26^, ^27^, ^28^, ^29^, ^30^, ^31^ More than ever diabetes guidelines are encouraging active personalizing of diabetes goals for glucose, blood pressure and cholesterol levels.^1^

We hypothesized that SDM with a decision aid tool that takes into account both treatment intensity, patient's clinical characteristics and patient's preferences could be effective in increasing the proportion of patients with T2DM who achieve all their personalized targets.^32^ We compared the results of multifactorial diabetes treatment after shared goal setting and prioritizing targets with a physician driven multifactorial diabetes treatment.

## 2 RESEARCH DESIGN AND METHODS

### 2.1 Study setting, practices and patients

The OPTIMAL study is an open cluster‐randomized controlled trial with a follow‐up of 24 months. It was not possible to blind participants and physicians for the treatment allocation. The full details of the rationale and design of this trial have been described previously.^32^ In short, the intervention included SDM with personalized goal setting and the use of a decision aid. Because SDM and goal setting are especially useful when there are at least two equally beneficial treatment options, the study was performed in primary care practices that participated in the ADDITION study between 2002 and 2009. The ADDITION study included screen detected patients with T2DM and compared an intensive multifactorial treatment with less intensive usual care according to national guidelines. The intensive treatment was associated with a significant increase in prescribed medications and a non‐significant 17% reduction of cardiovascular events and death after five years.^7^ The rate of cardiovascular events seemed to diverge after four years of follow‐up. It was concluded that intensified treatment and treatment according to national guidelines can theoretically be equally effective.^7^ In 2011/2012, all primary care practices that participated in the ADDITION study were invited to participate in the OPTIMAL study. Eligible practices were those familiar with the ADDITION‐protocol and which had included at least one patient in the ADDITION‐study.^32^ Randomization was executed at practice level at the research centre according to computer generated list independent of the study team, without any stratification. Practices were randomized a second time (1:1), that is, intervention practices in the ADDITION study could be control practices in this study and vice versa. To develop an intervention that should be implementable on a larger scale, the general practitioners (GPs) from the intervention group were trained in the SDM approach during just one 2‐hours training session, in which the study protocol, the SDM principles and the OPTIMAL decision aid were discussed.^32^ GPs were trained with role‐plays in the SDM process. All participating GPs included at least two more or less comparable patients: 1. Patients diagnosed with T2DM in 2002‐2004 by screening, aged between 50 and 70 years at that time and having participated in the ADDITION study; 2. Patients with T2DM not diagnosed in the ADDITION study, between 60 and 80 years in 2012‐2014 and with a T2DM duration between 8 and 12 years. Patients with a history of alcoholism, drug abuse, psychosis, personality disorder or another emotional, psychological or intellectual problem that is likely to invalidate informed consent, or limit the ability of the individual to comply with the protocol requirements were excluded. Also, patients with a limited life expectancy were excluded from participation.^32^

All eligible patients were approached, and informed consent was taken, after which they were invited for the first visit.

The study protocol was registered at the International trial registration (NCT02285881) and approved by the Medical Ethical Committee of the University Medical Centre Utrecht (Protocol number: 11‐153).

### 2.2 Patient involvement

At the end of the ADDITION study, all participating Dutch patients were invited to attend a meeting for the presentation of the 5‐year results. During that meeting with around 100 participants the idea arose to get the intensive treatment implemented in daily practice, but on the other hand patients stated that each individual should have the choice to choose the intensive or less intensive treatment option. During that meeting the idea for the OPTIMAL study came up. Later on, some patients were involved in the design of the decision aid. Patients were not involved by the recruitment and design of participants for the OPTIMAL study.

### 2.3 Intervention

#### 2.3.1 Theoretical framework

A theoretical framework for SDM in clinical practice was provided by Charles et al.^33^ They highlighted the need for bidirectional information exchange and agreement about the treatment. Originally this framework was developed for the acute setting; it was modified for chronic conditions in 2006.^3^ In chronic conditions, a long‐term relationship between clinicians and patients is essential, and the opportunity to revise decisions should be possible. The other components of the framework (partnership, information, deliberation and decision) remained similar to the original one.

#### 2.3.2 Decision support aid

The OPTIMAL decision support aid is a simple paper‐based tool, easy to use for both GP and the patient.^32^ It was used during the first visit to discuss 1) two treatment protocols; “usual care” vs intensified care, and 2) to prioritize five treatment targets (see below). Against that background, the decision aid consists of three steps: 1) considering the pros and cons of two almost equally effective evidence‐based multifactorial treatments, namely the intensified ADDITION protocol and the protocol derived from the Dutch guidelines for GPs^34^ followed by a shared decision on which protocol will be used; 2) prioritizing of treatment targets according to the chosen treatment protocol and 3) treatment selection (medication and/or lifestyle change); the way how to achieve the treatment targets.^32^ The same tool was used during the 12‐month follow‐up visit to reconsider the treatment priorities, not the intensity of treatment. Patients who were treated before the start of the study according to the Dutch guidelines could change their therapy to the intensified treatment, and patients who were treated intensively in the ADDITION study could alter their treatment to the less intensive option at baseline. So at the start of the OPTIMAL study all patients in the intervention group could change the intensity of their treatment or not.

#### 2.3.3 Control group

The GPs from the control practices were not asked to engage in SDM, nor trained to do so, and they were not offered the decision support aid. They were requested to treat the patients as they were used to since the ending of the ADDITION study (2009), either following the national guidelines or the ADDITION intensive treatment protocol, each with their respective targets. So patients in the control practices received treatment‐as‐before with their respective targets.^32^

#### 2.3.4 Treatment targets

Thresholds to start lowering the HbA1c‐level for the intensive treatment (derived from the ADDITION‐protocol) and according to the less intensive treatment (based on the Dutch guidelines) in order to reach treatment targets were 48 and 53 mmol/mol, respectively. With regard to the systolic blood pressure, these thresholds were 120 vs 140 mm Hg and for cholesterol levels 3.5 vs 4.5 mmol/L, respectively.

Treatment targets to be achieved during the trial for HbA1c were <53 mmol/mol in both treatment options, for systolic blood pressure ≤135 mm Hg (intensive) vs <140 mm Hg (less intensive) and—surprisingly—for cholesterol <5.0 vs <4.5 mmol/L. Besides the above mentioned thresholds and targets participants were recommended in both treatment options to stop smoking and in case of a BMI>25 to lose at least 5% of their body weight. Therefore, also weight and smoking status were considered treatment targets.

#### 2.3.5 Outcome measures and data collection

Data on patient characteristics were collected at baseline by patients self‐report on a case report form and included age, gender, education level, diabetes duration, living situation (alone or together) and smoking status. Data about medication, comorbidity, the shared choice for intensive or less intensive treatment, the prioritizing of the targets; and how to achieve the treatment targets (by medication and/or lifestyle changes) were reported on a separate case report form by the GP during visit 1 (baseline) and after 12 and 24 months.

HbA1c and total cholesterol, both at baseline and after 12 and 24 months, were analysed at the SHL Centre for Diagnostic Support in Primary Care, Etten‐Leur. HbA1c levels were analyzed with high‐performance liquid chromatography (Tosoh G8 machine) and total cholesterol levels with standard enzymatic techniques (Cobas 8000 machine).

Height and body weight were measured in light indoor clothing and without shoes using a fixed rigid stadiometer and a scale, respectively. Blood pressure was measured by two measurements after at least 10 minutes rest, while participants were seated with the cuff on the predominant arm at the level of the heart.^32^

### 2.4 Statistical analyses

Primary outcome was the proportion of patients that achieve all three treatment goals for HbA1c, blood pressure and total cholesterol after 24 months. To detect a difference of 20% between groups in the proportion of patients achieving all treatment targets,^7^ assuming a two‐sided significance level of 5%, with alpha 0.05 and power of 80% and with a dropout of 10% and a cluster effect of 1.125,^32^ a minimum number of 73 patients in each treatment group is required (Department of Statistics Sample Size Calculator, University of British Columbia).

Data were compared by group allocation, using either means (standard deviation, SD) or medians (interquartile range, IQR) for continuous variables and counts and percentages for nominal variables. The number of targets achieved at baseline was based on the source of recruitment (ADDITION Intensive, ADDITION Dutch Guidelines and non‐ADDITION). The treatment targets for the control group were assumed to be unchanged during the whole study period. Because it became clear that almost 90% of the participants did not smoke (anymore) and because in the control group there was no specific treatment target formulated for weight loss, we decided to analyze the proportion of patients that achieved treatment goals with respect to HbA1c, SBP and cholesterol levels.

Intention‐to‐treat analyses were performed to examine between‐group differences. To analyze the proportion of achieved treatment goals for all three treatment goals (blood pressure, lipids and HbA1c) relative risks and the number needed to treat (NNT) were calculated. Relative risks were assessed at 24‐months follow‐up for the complete cases (scenario 1), with the last observation carried forward (scenario 2), and as “targets not achieved” if the last measurement was missing (scenario 3). Generalized linear models were used to correct for clustering at practice level. A *P*‐value of <.05 is considered statistically significant. Two years differences within groups were analyzed using paired *t* tests, and differences between groups for Hba1c, total cholesterol, BMI and blood pressure were analyzed using ANCOVA with change scores. In the model, treatment allocation (intervention or control group) was included as factor and the baseline score as covariate. Differences within groups with respect to HbA1c, systolic blood pressure and total cholesterol were tested with paired *t* tests.

## 3 RESULTS

**Figure 1:**
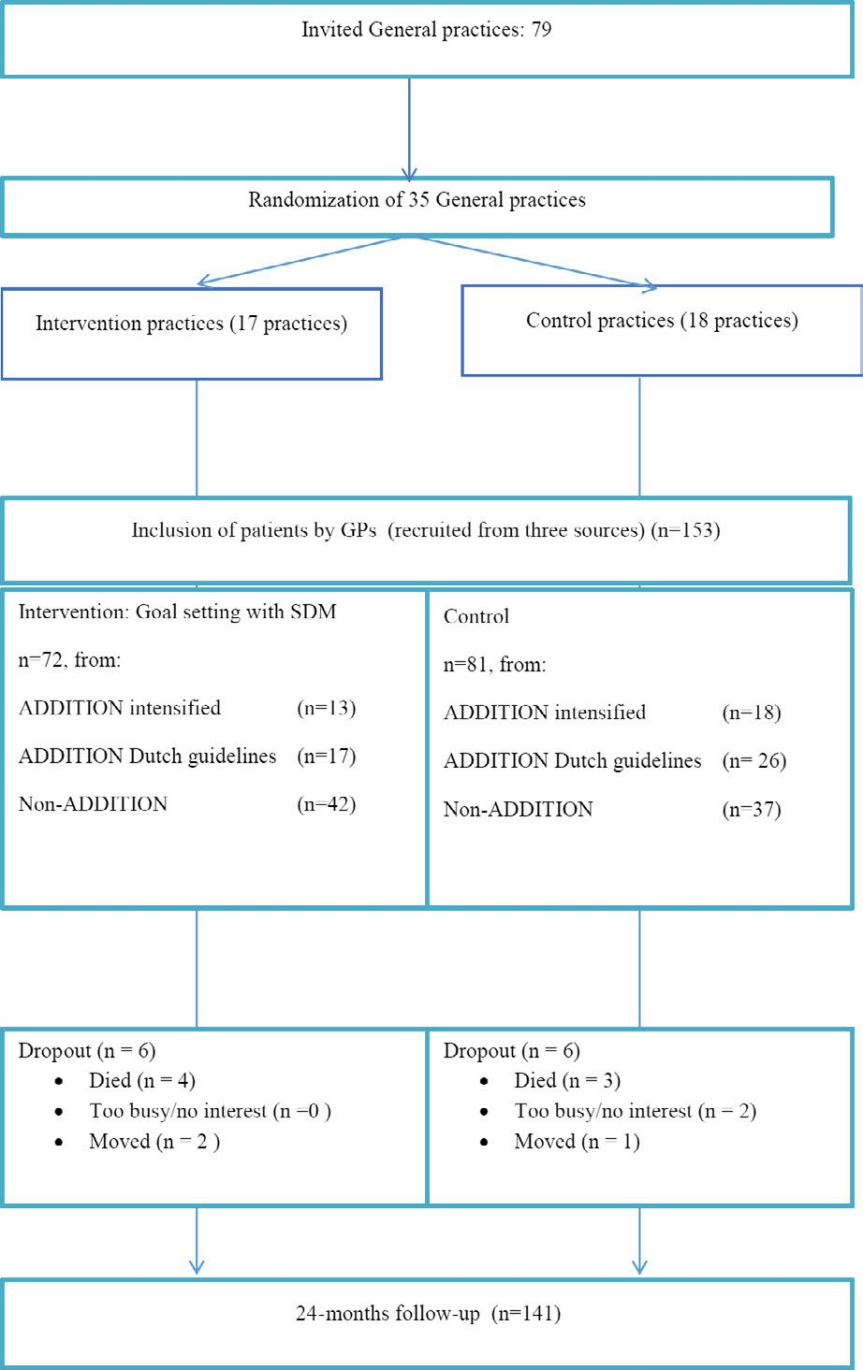
Flow diagram of patient enrollment, allocation and analysis

All 79 former ADDITION practices were invited, of which 35 practices agreed to participate (n=17 intervention and n=18 control group). From the original 435 ADDITION patients in these 35 practices, 74 patients could be included. Besides 79 more or less comparable non‐ADDITION patients were included. As a result, 153 patients were allocated to either the intervention or the control group (Figure 1). Overall, both groups were well matched, but fewer patients in the intervention group were treated with insulin or prescribed a statin (Table 1). During the study, seven participants deceased and four did not complete the final measurement. Dropout rates were similar in both groups (Figure 1).

**Table 1:** Baseline characteristics of participants in intervention and control group. Values are counts (percentages) unless stated otherwise

	Intervention (n=72)	Control (n=81)
Male gender	39 (54.2)	50 (58.8)
Age (years) mean (SD)	70.0 (5.7)	68.5 (5.7)
Duration of type 2 diabetes (years) mean (SD)	10.2 (2.3)	10.8 (3.5)
Education		
High	12 (16.7)	14 (17.3)
Middle	23 (31.9)	25 (30.9)
Low	37 (51.4)	42 (51.9)
Living alone	17 (24.2)	13 (15.6)
Current smoking	8 (11.1)	11 (12.9)
Body weight (kg) Mean (SD)	83.8 (14.8)	87.9 (13.4)
HbA1c (mmol/mol) Median (IQR)	49.0 (10)	50.5 (9)
Systolic blood pressure (mm Hg) Mean (SD)	138.1 (14.3)	137.2 (12.1)
Diastolic blood pressure (mm Hg) Median (IQR)	78 (10)	77 (10)
Total cholesterol (mmol/L) Median (IQR)	4.0 (1.2)	4.1 (1.0)
LDL—cholesterol (mmol/L) Median (IQR)	2.2 (1.2)	2.2 (0.8)
Medication		
Oral diabetes medication	61 (84.7)	70 (82.3)
Insulin	8 (11.1)	16 (18.8)
Statin	54 (75.9)	68 (80.0)
Other lipid regulating drugs	5 (6.9)	6 (7.0)
Use of blood pressure lowering drugs	60 (83.3)	72 (84.7)
Comorbidities		
Cardiac	15 (20.8)	15 (17.6)
Stroke	3 (4.2)	3 (3.5)
Chronic lung disease	5 (6.9)	5 (5.9)
Peripheral arterial disease	5 (6.9)	5 (5.9)

At baseline 26.4% of the 72 patients in the intervention group had achieved all treatment goals (control group: 23.5% of 81) (Table 2). After SDM 44 patients and their GP chose the intensive therapy: 10 of 13 patients continued their former intensive ADDITION therapy and 34 switched from less intensive to intensive. Twenty‐eight patients and their GP chose the less intensive protocol: 25 continued their former treatment and three people switched from the more intensive to the less intensive protocol. During the first visit 45.8% of participants prioritized weight loss, while blood pressure and glycaemic control were prioritized by 25.0% and 20.8%, respectively. These percentages hardly changed during the 12 and 24 months follow‐up visits. After 24‐months follow‐up the proportion of patients that achieved all three targets had increased in the intervention group from 26.4% to 31.8% (n=66), a relative improvement of 20%; it remained stable in the control group (25.3% (n=75)), with a NNT of 15.4 and a non‐significant relative risk of 1.26 (95% CI: 0.81‐1.95). If the last value was carried forward, 30.6% (n=72) and 24.7% (n=81) of the participants achieved all three treatment targets with a relative risk of 1.24 (95% CI 0.80‐1.90). Assuming that dropouts did not achieve all three treatment targets, percentages were 29.2% (n=72) and 23.5% (n=81), respectively, with a relative risk of 1.24 (95% CI: 0.73‐2.12). After adjustment for practice level, patients in the intervention group still reached more often all three treatment goals, although the intervention effect was not significant (regression coefficient 0.277, *P*=.71). The proportion of participants that achieved two treatment goals (all combinations) after 24 months was similar in both groups (39.4% in the intervention and 37.3% in the control group) (Table 3). Seven participants in the intervention group and eight participants in the control group achieved all treatment goals both at baseline and after 24 months. None of the participants achieved all three treatment goals after 24 months if at baseline none of the targets was achieved. All patients who achieved three treatment goals at baseline achieved at least one target at follow‐up. Four patients in the intervention group had achieved one goal at baseline and achieved all three treatment goals after 24 months, and one participant achieved the opposite. In the control group, these numbers were three and two participants, respectively. Four participants in the intervention group (control group: two) did not achieve any goal during the study period. From all the treatment goals, the target for total cholesterol was most often met in both groups (80.3% vs 68%, respectively, (*P*=.076). Blood pressure decreased significant only in the intervention group (−5.4 mm Hg, *P*<.01). Mean HbA1c, total cholesterol and BMI did not change during follow‐up in either group. Between‐group differences were not significant (Table 4).

**Table 2:** Numbers and percentages of participants who achieved targets for HbA1c, SBP and total cholesterol at baseline and at 24 months

	Intervention		Control	
	Baseline (n=72)	Follow‐up (n=66)^a^	Baseline (n=81)	Follow‐up (n=75) a
HbA1c	49 (68.1)	38 (57.6)	46 (56.8)	38 (50.7)
		39 (54.2)		40 (49.4)
		38 (52.8)		38 (46.9)
Systolic blood pressure	37 (51.4)	43 (65.2)	35 (43.2)	46 (61.3)
		46 (63.9)		50 (61.7)
		43 (59.7)		46 (56.8)
Total cholesterol	50 (69.4)	53 (80.3)	54 (66.7)	51 (68.0)
		55 (76.4)		54 (66.7)
		53 (69.4)		51 (63.0)
All three treatment targets	19 (26.4)	21 (31.8)	19 (23.5)	19 (25.3)
		22 (30.6)		20 (24.7)
		21 (29.2)		19 (23.5)

a Numbers and percentages after 24 months in case of complete cases (scenario 1), as last observation carried forward (scenario 2), and as “not achieved” if the last measurement was missing (scenario 3).

**Table 3:** Number of people (%) achieving 0‐3 targets after 24 months, specified for specific targets and study group

Number of targets	HbA1c	SBP	Total Cholesterol	Number (%) of patients	All patients
Intervention group (n=66)				Intervention group	
n=3				21 (31.8)	21 (31.8)
n=2				3 (4.5)	
n=2				12 (18.2)	26 (39.4)
n=2				11 (16.7)	
n=1				3 (4.5)	
n=1				3 (4.5)	15 (22.7)
n=1				9 (13.6)	
n=0				4 (6.0)	4 (6.0)
	38 (57.6)	43 (65.2)	53 (80.3)		
Control group (n=75)				Control group	
n=3				18 (24.0)	19 (25.3)
n=2				8 (10.7)	
n=2				9 (12.0)	28 (37.3)
n=2				11 (14.7)	
n=1				5 (6.7)	
n=1				9 (12.0)	26 (34.7)
n=1				12 (16.0)	
n=0				2 (2.7)	2 (2.7)
	38 (50.0)	46 (60.5)	51 (67.1)		

**Table 4:** HbA1c, SBP, total cholesterol and BMI both at baseline and after 24 months. Means (SD) and *P*‐values within and between groups

	Intervention			Control					
	Baseline	2 years	*P*‐value^a^	Baseline	2 years	*P*‐value^a^	F	Mean difference^b^	*P*‐value
HbA1c (mmol/mol)	50.7 (9.6)	52.9 (11.1)	.07	51.6 (9.0)	51.8 (7.0)	.69	2.3	2.15	.14
SBP (mm Hg)	138.1 (14.3)	132.7 (15.3)	<.01	137.2 (12.1)	135.7 (12.2)	.11	2.1	−3.3	.15
Total cholesterol (mmol/L)	4.2 (1.0)	4.2 (1.0)	.98	4.3 (1.0)	4.2 (0.9)	.09	0.84	0.13	.36
BMI	29.6 (3.8)	29.4 (4.0)	.48	30.1 (4.5)	30.0 (4.4)	.53	1.71	−0.07	.82

^a^Represents the results of the within group differences (paired *t* test) and ^b^the results of the between‐group differences (ANCOVA, adjusted for baseline value).

## 4 CONCLUSIONS

This study shows that by taking into account both patients' treatment preferences and making shared decisions resulted in a higher proportion of people who achieved all their treatment goals, whereas it did not change in the control group. However, the difference between groups did not reach significance, which is possibly the result of a higher, namely 24.8% (38/153) instead of the assumed 10% proportion of participants that already had achieved all three treatment goals at baseline. The relative improvement was about 20% in the intervention group. Our primary outcome measure was based on intermediate endpoints, which is necessary to convince physicians to implement the SDM‐goal setting approach within chronic care.^4^ From a Cochrane review it became clear that the use of a decision aid resulted in a significant improvement in more accurate perceptions of health outcome probabilities, and more congruence between the chosen options and the person's values.^25^, ^35^ We think it is important that in the OPTIMAL study the treating physician presented comparative evidence to the patient of two multifactorial treatment protocols and could demonstrate the possible impact of treatment intensity on cardiovascular morbidity and mortality.

Weight loss was the highest priority of most patients, both at baseline and after 12 and 24 months. However, mean body weight did not change over time (baseline to 24 months) in both groups. The direct effectiveness of weight loss on both intermediate and cardiovascular outcome could also have been presented to the patient, which might have been helpful in achieving the targets in this respect. To further increase the proportion of patients who achieve their treatment targets it is suggested to write the shared goals on a specific form, not only to register in the medical records for the physician (as is done in OPTIMAL), but also for the patient to take home. Another suggestion is to discuss the agreed targets during 3‐monthly check‐ups with the practice nurse,^4^, ^20^, ^21^ instead of only during the annual check‐ups with the physician (as is done in OPTIMAL).

One might argue that our decision aid was based upon a study with treatment options that do not differ largely.^25^ In a meta‐analysis the pooled effect of personalized care planning with SDM‐goal setting showed a small decrease of HbA1c of −0.24% (−0.35 to −0.14) and a −2.64 mm Hg (−4.47 to −0.82) decrease in systolic blood pressure.^4^ Compared to these results a larger decrease in SBP was found in the current study, but less in HbA1c. This result is not surprising considering the already low baseline levels of HbA1c.

Strength of the current study is that in the SDM process the treating physician could present evidence with a direct relation between intensity of treatment and so‐called “hard outcome”. Furthermore, the patient's usual diabetes care provider performed the SDM‐goal setting approach, which is an essential element in the context of chronic conditions, while this was not performed in the control practices (data not shown). The follow‐up time of 24 months with yearly recalibration of chosen goals reflects changes in conditions and side‐effects of interventions within chronic care. With a follow‐up time of 24 months in 35 general practices with 208 intervention consultations, we had a real pragmatic trial, which is also reflected by the opinion of three quarters of the GPs in the intervention practices who find the decision aid useful for shared decision and annual use, although it took a little extra time (data not shown). In this respect, it seems feasible to integrate the decision aid, as it is expected that once the GP is used to work with the decision aid no extra time is needed. However, if the decision aid will be implemented, it is imported to keep it up to date. In contrast to most RCTs, in the current study the percentage of participants with a high education was relatively low (17% high vs. 40% low educated), and therefore, more representative for the average population with T2DM.

However, several limitations should also be considered. For an optimal connection between evidence‐based medicine and SDM in our intervention, the physician should have presented all available evidence to the patient with regard to the effectiveness of multifactorial diabetes treatment on cardiovascular outcomes. Given the diabetes duration in our study population of more than 10 years on average, the results of the STENO‐2 study could have been included in the decision aid.^6^ In this study in people with known type 2 diabetes and with microalbuminuria, with a median diabetes duration of 6 years at baseline, and intensified multifactorial treatment resulted in 20% absolute risk reduction in mortality after 13.3 years follow‐up.^6^ Our decision aid did not mention explicitly how individual characteristics like age, diabetes duration or comorbidity had to be taken into account during the SDM process with regard to the intensity of the multifactorial treatment. However, the way to achieve treatment targets was part of the SDM process acknowledging the clinicians' medical knowledge, the social context of the patient and the patients' preferences. If health‐care providers should communicate with their patients regarding suitable treatment targets, treatment strategies and alternatives options, risks and benefits and potential side‐effects, this might lessen the chance of clinical inertia.^36^ Finally, we should realize that in SDM it is also important to set emotional and social management goals.^21^ In our intervention, we did not measure this type of goals, which could be considered as a drawback.

To conclude, taking into account both patients' preferences with regard to the intensity of treatment and his/her priorities resulted in a higher, but not significant, proportion of people who achieve all treatment goals after two years. In this pragmatic trial in a substantial number of general practices with an already existing high baseline level of diabetes care, we found indications that SDM on both intensity of treatment and prioritizing treatment goals could lead to a further improvement of diabetes care.

## CONFLICT OF INTEREST

The authors declare that they have no competing interests.

## AUTHOR'S CONTRIBUTION

HdO collected and analyzed data and wrote the manuscript. RV analyzed the data, reviewed and edited the manuscript. GR designed the study, reviewed and edited the manuscript. GR (the manuscript's guarantor) affirms that the manuscript is an honest, accurate and transparent account of the study being reported; that no important aspects of the study have been omitted; and that any discrepancies from the study as planned (and, if relevant, registered) have been explained. All authors had full access to all of the data (including statistical reports and tables) in the study and can take responsibility for the integrity of the data and the accuracy of the data analysis.
