# Supplementary figures and images for: Effectiveness of shared goal setting and decision making to achieve treatment targets in type 2 diabetes patients: A cluster‐randomized trial (OPTIMAL)
Source: Health Expect. 2017 May 24;20(5):1172–80. doi: 10.1111/hex.12563 (PMC5600211; doi:10.1111/hex.12563)

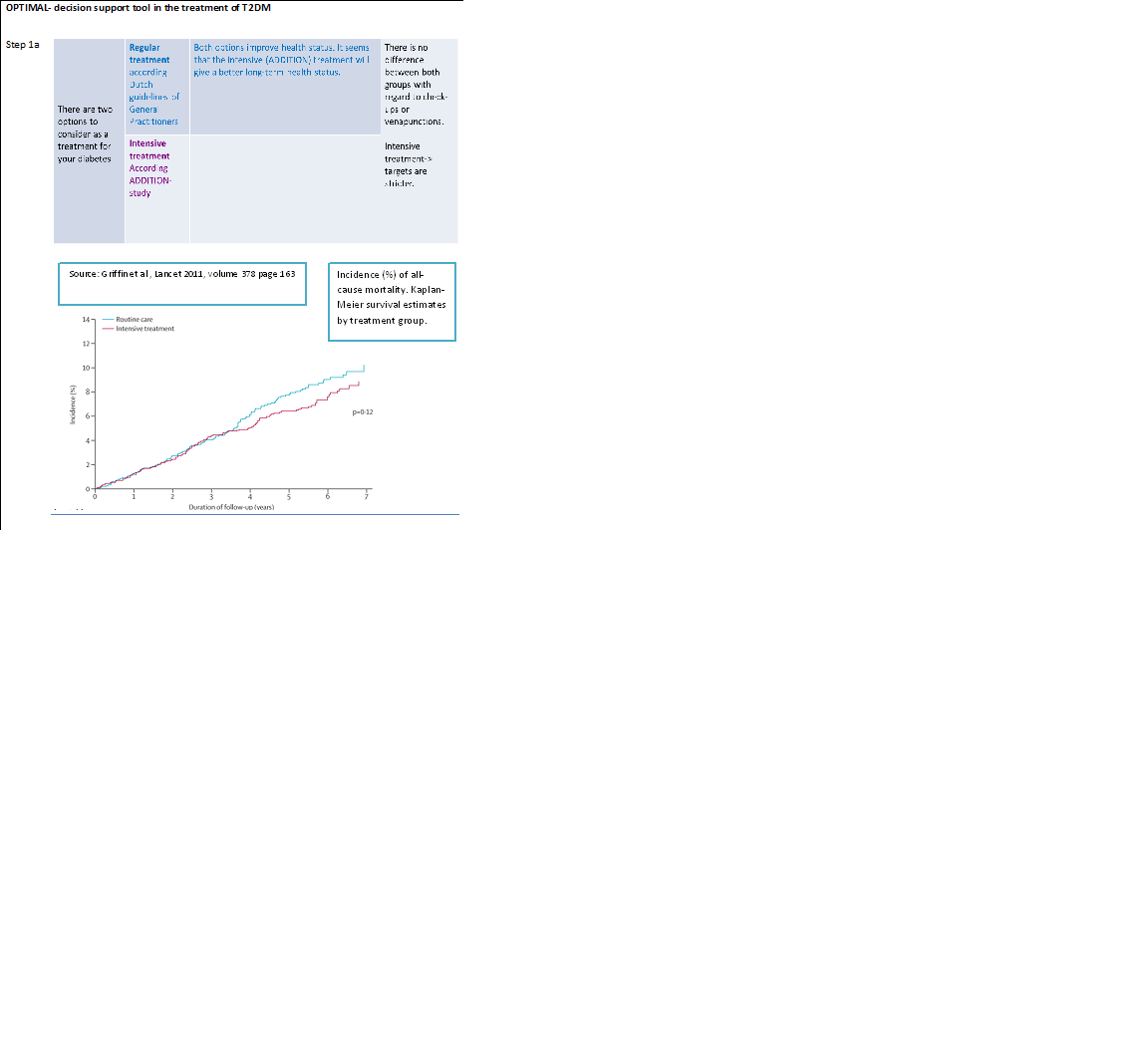

Supplement: Supplementary file 1 [file HEX-20-1172-s001.tif]

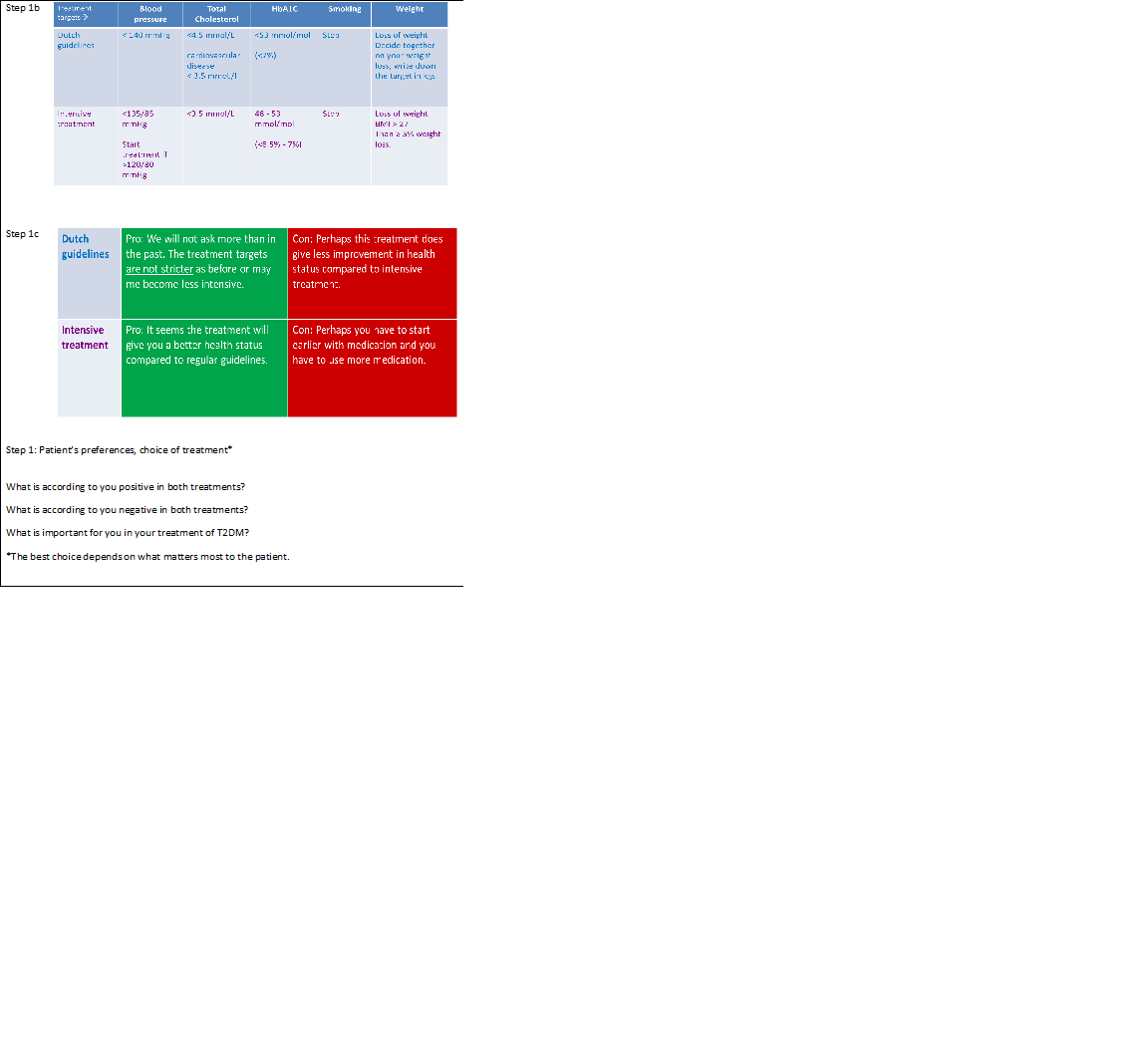

Supplement: Supplementary file 2 [file HEX-20-1172-s002.tif]

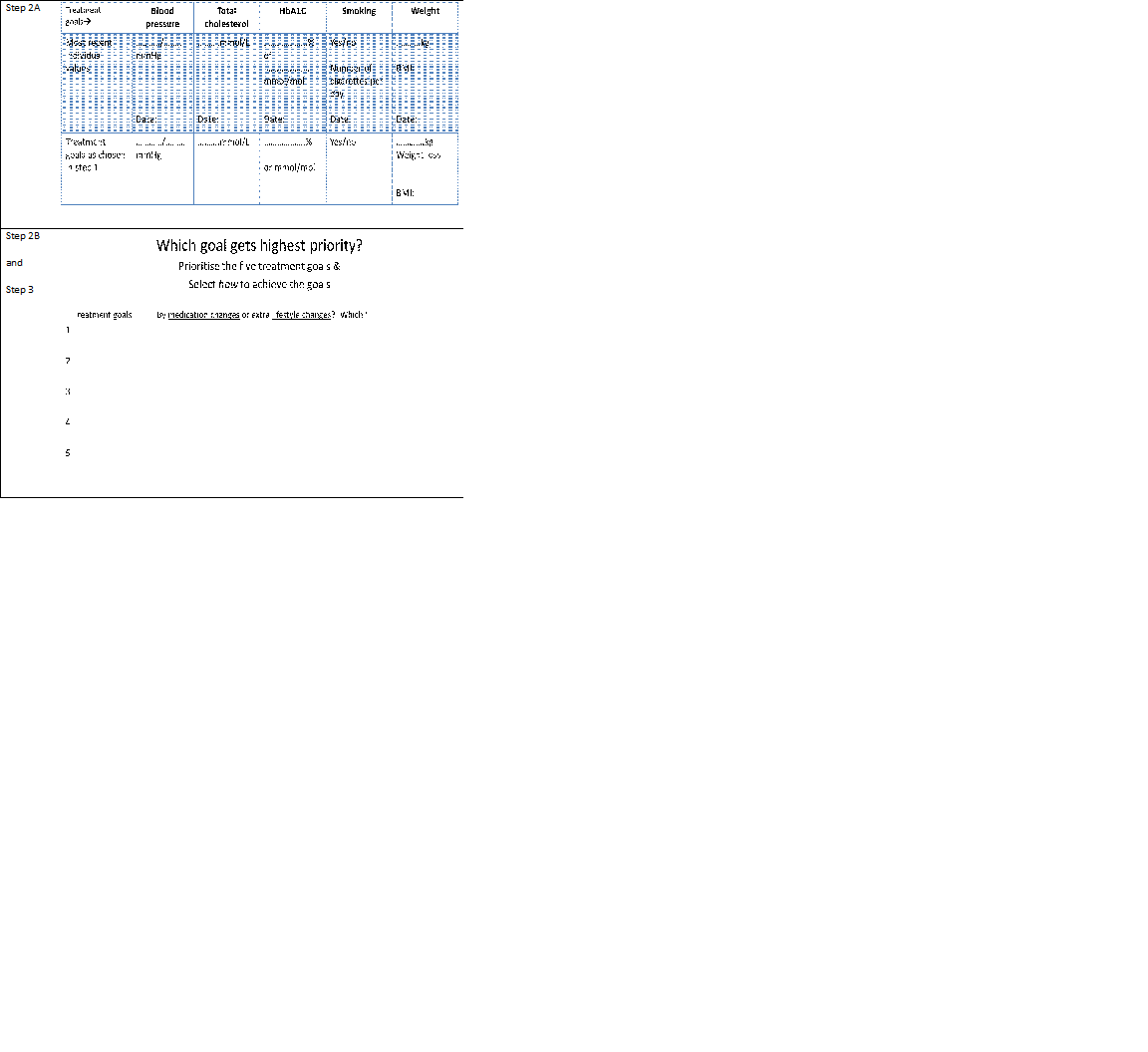

Supplement: Supplementary file 3 [file HEX-20-1172-s003.tif]
